# Cyclic Stretch Induces Vascular Smooth Muscle Cells to Secrete Connective Tissue Growth Factor and Promote Endothelial Progenitor Cell Differentiation and Angiogenesis

**DOI:** 10.3389/fcell.2020.606989

**Published:** 2020-12-09

**Authors:** Jing Yan, Wen-Bin Wang, Yang-Jing Fan, Han Bao, Na Li, Qing-Ping Yao, Yun-Long Huo, Zong-Lai Jiang, Ying-Xin Qi, Yue Han

**Affiliations:** School of Life Sciences and Biotechnology, Institute of Mechanobiology and Medical Engineering, Shanghai Jiao Tong University, Shanghai, China

**Keywords:** cyclic stretch, endothelial progenitor cells, differentiation, angiogenesis, vascular smooth muscle cells, connective tissue growth factor

## Abstract

Endothelial progenitor cells (EPCs) play a vital role in endothelial repair following vascular injury by maintaining the integrity of endothelium. As EPCs home to endothelial injury sites, they may communicate with exposed vascular smooth muscle cells (VSMCs), which are subjected to cyclic stretch generated by blood flow. In this study, the synergistic effect of cyclic stretch and communication with neighboring VSMCs on EPC function during vascular repair was investigated. *In vivo* study revealed that EPCs adhered to the injury site and were contacted to VSMCs in the Sprague–Dawley (SD) rat carotid artery injury model. *In vitro*, EPCs were cocultured with VSMCs, which were exposed to cyclic stretch at a magnitude of 5% (which mimics physiological stretch) and a constant frequency of 1.25 Hz for 12 h. The results indicated that stretched VSMCs modulated EPC differentiation into mature endothelial cells (ECs) and promoted angiogenesis. Meanwhile, cyclic stretch upregulated the mRNA expression and secretion level of connective tissue growth factor (CTGF) in VSMCs. Recombinant CTGF (r-CTGF) treatment promoted endothelial differentiation of EPCs and angiogenesis, and increased their protein levels of FZD8 and β-catenin. CTGF knockdown in VSMCs inhibited cyclic stretch-induced EPC differentiation into ECs and attenuated EPC tube formation via modulation of the FZD8/β-catenin signaling pathway. FZD8 knockdown repressed endothelial differentiation of EPCs and their angiogenic activity. Wnt signaling inhibitor decreased the endothelial differentiation and angiogenetic ability of EPCs cocultured with stretched VSMCs. Consistently, an *in vivo* Matrigel plug assay demonstrated that r-CTGF-treated EPCs exhibited enhanced angiogenesis; similarly, stretched VSMCs also induced cocultured EPC differentiation toward ECs. In a rat vascular injury model, r-CTGF improved EPC reendothelialization capacity. The present results indicate that cyclic stretch induces VSMC-derived CTGF secretion, which, in turn, activates FZD8 and β-catenin to promote both differentiation of cocultured EPCs into the EC lineage and angiogenesis, suggesting that CTGF acts as a key intercellular mediator and a potential therapeutic target for vascular repair.

## Introduction

Vascular injury leads to the initiation and progression of atherosclerotic vascular disease and may result in neointimal hyperplasia, in-stent restenosis, and acute stent thrombosis (Deanfield et al., 2007). Therefore, regeneration of the vascular endothelium is very important. Due to a low proliferative ability, the capacity of mature endothelial cells (ECs) to replace damaged endothelium is limited during vascular repair (Hristov et al., 2003; Deanfield et al., 2007). Accumulating studies indicate that bone marrow-derived endothelial progenitor cells (EPCs) play a crucial role in maintaining endothelial integrity. When damage occurs, EPCs are mobilized and home to the injury sites and then differentiate into ECs, which participate in angiogenesis, neovascularization, and tissue repair (Asahara et al., 1997; Deanfield et al., 2007; Zhang et al., 2014; Li et al., 2019). In this process, EPCs communicate with the exposed vascular smooth muscle cells (VSMCs), which compose the medial layer of the vessel wall. However, the impact of VSMCs on EPC function or the underlying signal mechanisms is little known.

Many studies have shown that EC and VSMC interaction in hemodynamic environments participates in the regulation of vascular stabilization, remodeling, and function, which suggests a role for mechanical forces generated by the pulsatile nature of blood pressure and flow in these processes (Chien, 2007; Qi et al., 2011; Chen et al., 2013; Deng et al., 2015). For example, VSMCs secrete exosomes enriched with miR-143-3p that are transported to ECs, and endothelial proliferation is subsequently induced under pathologically elevated cyclic stretch (Deng et al., 2015). Mesenchymal stem cells promote the differentiation of EPCs into ECs via secreting VEGF and increasing expression of CD31 and vWF (Ge et al., 2018). These studies indicated that the cellular microenvironment and the resident cells might participate in determining EPC differentiation fate. Under physiological conditions, VSMCs are constantly subjected to mechanical stretching *in vivo*; therefore, we aimed to investigate whether mechanical forces and neighboring VSMCs modulate the endothelial differentiation of EPCs during endothelium repair.

Several studies have revealed that EPCs differentiate into either myocardial cells or endothelial cells, related to the cellular microenvironment of cytokines and coexisting cells (Murasawa et al., 2005; Ge et al., 2018). Resident cells contribute to the vascular repair process mostly via paracrine processes, secreting a mixture of growth factors and cytokines to recruit numerous cells to the injured sites and modulate the cell functions (Armulik et al., 2005; Ostriker et al., 2014). Connective tissue growth factor (CTGF), also known as CCN2, is a secreted protein of the CCN family, which includes Cyr61, CTGF, NOV, WISP1, WISP2, and WISP3 (Kubota and Takigawa, 2007; Yu et al., 2010; Jun and Lau, 2011; Liu et al., 2014). It has been reported that CTGF is implicated in vascular diseases, including atherosclerosis, hypertension, restenosis, and thrombosis. CTGF promotes neointimal hyperplasia after vascular injury and in rupture-prone atherosclerotic plaques; especially in areas of neovascularization and in the neointima in restenosis after balloon injuries, high CTGF expression levels are detected (Cicha et al., 2005; Kundi et al., 2009). It was also found that CTGF was strongly expressed in vascular cells, such as VSMCs, ECs, and fibroblasts (Jun and Lau, 2011). In our previous study, we found that CTGF is upregulated both *in vivo* and *in vitro* during hypertension, and 15% cyclic stretch significantly increased CTGF expression in VSMCs via microRNA-19b-3p, which indicated that CTGF is a key mechanical-sensitive molecule (Wang et al., 2019). Moreover, CTGF also plays a significant role in cell growth and differentiation. CTGF and VEGF induced pluripotent stem cells to differentiate into mature ECs (Kelly et al., 2020), and adipose-derived stem cells can differentiate and proliferate with CTGF stimulation to enhance tendon repair (Li et al., 2019). However, the relationship between CTGF and EPCs has not been widely concerned and studied. Therefore, we hypothesized that VSMC-derived CTGF contributes to the endothelial differentiation of EPCs in the process of vascular repair.

In this study, a rat vascular injury model and *in vivo* Matrigel plug assay were established to explore the effect of CTGF on EPC differentiation during vascular repair. An *in vitro* EPC//VSMC coculture system that included cyclic stretch was developed to detect the intercellular relationship between them. Here, we aimed to investigate whether CTGF is a key cell-to-cell interaction regulator for EPC differentiation in vascular repair.

## Materials and Methods

### Rat Carotid Artery Intimal Injury Model and Reendothelialization Assay

Sprague–Dawley (SD) rats were anesthetized with isoflurane, and then the carotid arteries were exposed under the anatomical microscope. After the bifurcation above the common carotid artery was found, the occipital artery, the internal carotid artery, the thyroid artery, and the external carotid artery were ligated with a surgical suture in turn, and the thoracic segment of the common carotid artery was clamped with a hemostatic forceps. A balloon inserted (0.67 mm; Edwards Lifesciences, CA, USA) was applied to damage the intima through a small opening from the proximal end of the thyroid artery. EPCs (1 × 10^6^) were pretreated with CM-Dil (1 μM, YEASEN, Shanghai, China) for 5 min at 37°C for labeling cells in red. Then, the cells were resuspended in 200 μl of PBS and the suspension was instilled into and incubated with the freshly injured arterial bed for 25–30 min (Griese et al., 2003; Yan et al., 2020). Postoperative rats continue to be housed with the experimental animal center. Recombinant CTGF (2 μg/kg/day) was firstly injected from the tail vein 3 h after injury and continued for 7 days. To observe the adhesion of EPCs, the left common carotid arteries were collected from the carotid bifurcation, incised longitudinally, and flattened between coverslips. The samples were fixed with 4% paraformaldehyde for 24 h and permeabilized with 0.3% Triton X-100 for 30 min. Nucleus was marked with DAPI (1:1,000) for 15 min at room temperature, and confocal microscopy (LV1000; Olympus, Tokyo, Japan) was used to take and analyze fluorescent images. Evans blue (2%, 40 mg/kg) was injected into the tail vein 30 min before sacrifice for the detection of reendothelialization assay on the seventh day after intimal injury. Photographs were taken with a digital camera. We quantified the reendothelialized parts that defined as areas not stained in blue with ImageJ software (Yan et al., 2020).

### Cell Culture and Identification

VSMCs were harvested from the thoracic aorta of male SD rats by an explant technique (Qi et al., 2008, 2010). VSMCs were cultured in Dulbecco's modified Eagle medium (DMEM, Gibco) containing 10% fetal bovine serum (FBS, Gibco) and incubated at 37°C with 5% CO_2_. These cells were characterized by immunofluorescent staining, which was performed with an antibody recognizing smooth muscle specific α-actin (Sigma). VSMCs from passages 4 to 8 were used in the following study.

EPCs derived from the bone marrow of SD rats were isolated and cultured as reported (Kuliszewski et al., 2009; Li et al., 2012). Briefly, total mononuclear cells were isolated from the tibias and femurs of male SD rats by density-gradient centrifugation with Histopaque-1083 (Sigma); the cells were then cultured in six-well plates with endothelial growth medium-2 (EGM-2) (Lonza) supplemented with fetal bovine serum (FBS), vascular endothelial growth factor (VEGF), fibroblast growth factor-B, epidermal growth factor, hydrocortisone, R3 insulin-like growth factor-1 (R3 IGF-1), ascorbic acid, and GA-1000 at 37°C in a 5% CO_2_ incubator. The medium was changed after 4 days, and subsequent media changes were made every 3 days.

EPCs were identified by classical method (Asahara et al., 1997; Wei et al., 2015). The cells were incubated with FITC-UEA-1 (Sigma) and Dil-Ac-LDL (Molecular Probes) as described previously. Double-positive staining for Dil-Ac-LDL and FITC-UEA-I was considered an indication of EPCs. Surface markers for flow cytometry analysis include the stem cell markers CD133 and CD34, as well as the endothelial marker CD31. EPCs were stained with a panel of monoclonal antibodies including anti-CD133 (1:100, Proteintech), anti-CD34-PE (1:100, eBioscience), anti-CD31-PE-Cy7 (1:100, eBioscience), and anti-CD45-PE (1:100, eBioscience). EPCs stained with anti-CD133 were incubated with an FITC-conjugated anti-mouse antibody. Unstained cells were incubated in parallel with antibody immunoglobulin (IgG) controls. Cells that were positive for CD133, CD34, and CD31 and negative for CD45 were considered to be EPCs. All samples were analyzed by flow cytometry (Becton Dickinson). EPCs from passages 2 and 3 were used (Li et al., 2018).

### EPC//VSMC Coculture Model and Cyclic Stretch Application

EPCs (1 × 10^5^ cells per well) were seeded on the inner side of a cell culture insert (Becton Dickinson), which has a 10-μm-thick polyethylene terephthalate (PET) membrane containing 0.4-μm pores. Flexible silicone bottom plates (Flexcell International) were either kept unseeded (EPC//O static group) or seeded with VSMCs (2 × 10^5^ cells per well, EPC//VSMC static group). The insert with EPCs was incorporated into the flexible silicone bottom plates, which were loaded with an FX-5000T strain unit system (Flexcell International) for the application of cyclic stretch at 5% stretch magnitude and 1.25 Hz frequency (Qi et al., 2016) for 6 or 12 h (EPC//VSMC stretch group) to mimic arterial mechanical conditions, as illustrated in **Figure 2A**.

### Western Blotting

Western blotting was performed as described previously (Qi et al., 2008). Proteins were separated by 10% SDS-PAGE and transferred to PVDF membranes (Millipore, 0.22 μm). The membranes were incubated overnight with primary antibodies, respectively, against CTGF (1:800, Proteintech), FZD8 (1:1,000, Abcam), β-catenin (1:1,000, Cell Signaling Technology), Lamin A (1:1,000, Abcam), and GAPDH (1:1,000, Proteintech) at 4°C, which was followed by immunoblotting with anti-rabbit or anti-mouse IgG (1:2,000, Cell Signaling Technology) conjugated to HRP. Protein bands were detected by a standard enhanced chemiluminescence (ECL; Tanon) method and quantified by Quantity One software (Bio-Rad). Nuclear extracts were prepared by the Nuclear Extraction kit (Beyotime) according to the manufacturer's instructions.

### Quantitative Real-Time Polymerase Chain Reaction (QPCR)

Total RNA was extracted from EPCs using Trizol reagent (Invitrogen) and was followed by cDNA synthesis using a RevertAid First Strand cDNA kit (Thermo Fisher Scientific). The resulting cDNA was used as a template in quantitative real-time polymerase chain reactions with SYBR Green Supermix on an ABI Prism 7500 sequence detection PCR system (Applied Biosystems) according to the manufacturer's protocol. The specific primer sequences are listed in Table 1. PCR conditions were as follows: 95°C for 30 s followed by 40 cycles at 95°C for 5 s, 60°C for 45 s, and 72°C for 30 s. The results were normalized to the GAPDH expression level, and relative quantification of the ratios was performed using the 2^−ΔΔCt^ method.

**Table 1:** The sequences of primers.

**Gene**	**Primer sequences (5**^**′**^**-3**^**′**^**)**	
CD31	Fwd: GACAGCCAAGGCAGAT GCAC	Rev: ATTGGATGGCTTGGCC TGAA
vWF	Fwd: GCGTGGCAGTGG TAGAGTA	Rev: GGAGATAGCGG GTGAAATA
KDR	Fwd: GGCACCACTCAAA CGCTGAC	Rev: CCTCTCTCCTCTC CCGACTT
CTGF	Fwd: GGAAATGCTGTGAGG AGTGGGTGT	Rev: TGTCTTCCAGTCGGT AGGCAGCTA
GAPDH	Fwd: GGCACAGTCAAGG CTGAGAAT	Rev: ATGGTGGTGAAGA CGCCAGTA

*Fwd, forward; Rev, reverse*.

### Transfection of Small Interfering RNA

The mRNA sequences of rat CTGF (NM_022266) were acquired from NCBI GenBank. Small interfering RNA (siRNA) against rat CTGF was designed and synthesized by GenePharma Biological Company (Shanghai, China). The sequences of the CTGF siRNAs were 5′-GGU CAA GCU GCC CGG GAA ATT-3′ and 5′-UUU CCC GGG CAG CUU GAC CTT-3′. The sequences of the FZD8 siRNAs were 5′-GGA AGU GAC CUC GCU ACU ATT−3′ and 5′-UAG UAG CGA GGU CAC UUC CTT -3′.

For the RNA interfering experiment, VSMCs were transfected with 100 nM specific siRNA or scrambled siRNA with 5 μl of Lipofectamine™ 2000 (Invitrogen) in Opti-MEM (Gibco) for 48 h following the manufacturer's instructions.

### Matrigel Tube Formation Assay

An *in vitro* tube formation assay was performed as previously described (Malinda, 2009). In brief, Matrigel (Corning) was added to 24-well plates at 300 μl per well and to polymerize at 37°C for 30 min. Then, 2 × 10^4^ EPCs were seeded onto the coated wells and cultured with 500 μl EBM-2 at 37°C with 5% CO_2_ for 6 h. EPC tube formation was assessed by microscopy, and photographs were randomly taken of five fields. The total tube length was calculated using imaging analysis software (Image-Pro Plus 6.0).

### *In vivo* Matrigel Plug Angiogenesis Assay

An *in vivo* angiogenesis assay was performed as previously described (Han et al., 2016). EPCs were treated with or without recombinant CTGF (r-CTGF; 20 ng/ml) for 24 h. Growth factor-reduced Matrigel (400 μl/plug) was thawed and mixed with EPCs (1 × 10^6^/plug), and Matrigel supplemented only with r-CTGF in the absence of EPCs was used as a blank control. In addition, to investigate how VSMCs and stretch affect EPC differentiation, EPCs were cultured by themselves under static conditions or cocultured with VSMCs, which had cyclic stretch applied at 5% stretch magnitude and 1.25 Hz frequency. After 12 h, EPCs under different conditions were harvested by treatment with 0.25% trypsin and were mixed with Matrigel. Each plug was subcutaneously injected into the flank of 6-week-old male nude mice (*n* = 4 in each group). Mice were euthanized 7 days after surgery. Matrigel plugs were harvested, fixed in 10% formalin, and embedded in paraffin; multiple 5-μm-thick slices were prepared. Immunofluorescent staining was performed using an anti-CD31 antibody (Abcam) and anti-CD34 antibody (Santa Cruz).

### Enzyme-linked Immunosorbent Assay (ELISA)

The concentration of CTGF in cell culture medium from VSMCs was determined by enzyme-linked immunosorbent assay by using a rat CTGF ELISA kit (Cloud-Clone Corp). The assay was performed according to the manufacturer's instructions.

### Ingenuity Pathway Analysis

Ingenuity Pathway Analysis (IPA) software (Qiagen) was used to search for the genes that possibly interact with CTGF. The software predicted the downstream genes of specific target through Grow Tool. After prediction, the possible canonical pathways and functional classifications of the target genes of CTGF were obtained with IPA. The significance values for analyses of network and pathway generation were calculated using the right-tailed Fisher's Exact Test by comparing the number of proteins that participate in a given function or pathway relative to the total number of occurrences of these proteins in all functional/pathway annotations stored in the Ingenuity Pathway Knowledge Base (IPKB). IPA was used to understand the complex biological and chemical systems at the core of life science research based on lectures or predicated analysis (Dai et al., 2009).

### Statistical Analysis

Data are expressed as the mean ± *SD* from at least three independent experiments. Statistical analysis was performed with Student's *t-*tests for two groups of data and with one-way ANOVA for multiple comparisons, followed by Bonferroni's *post-hoc* test. Non-parametric data were analyzed with the Mann–Whitney *t-*test in GraphPad Prism software. Values of *P* that were < 0.05 were considered statistically significant.

## Results

### The Adhesion of EPCs at Intimal Injury Site *in vivo*

**Figure 1:**
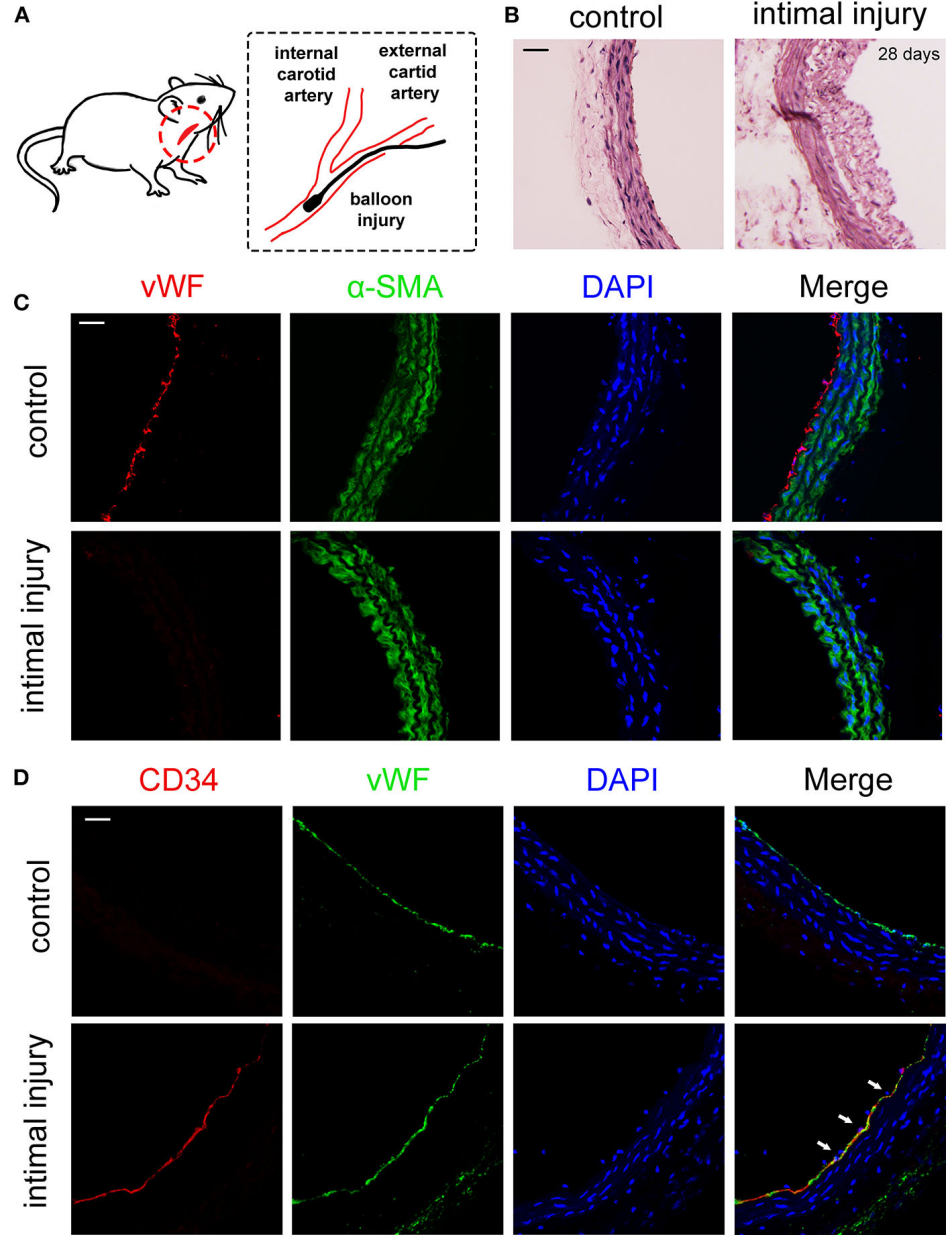
EPCs adhered to the injury site 1 h after balloon damaged the intimal. **(A)** Schematic diagrams of the establishment of rat intimal balloon damage model. **(B)** HE staining indicated the significant intimal hyperplasia and the thickening blood vessel wall after 28 days compared with control group (*n* = 4). **(C)** We harvested the vessels for the immunofluorescence staining right after the surgery. The immunofluorescence staining results showed that vWF (red) was not expressed after the balloon injury, indicating that the intima fell off and the model was successfully constructed. The α-SMA (green) and DAPI (blue) were used to identify VSMCs and nuclei, respectively (*n* = 4). Scale bar = 50 μm. **(D)** The adhesion of EPCs *in situ* 1 h after intimal injury was identified by CD34 (stem cell marker, red) and vWF (EC marker, green) by immunofluorescence staining. The results showed that CD34 and vWF were co-expressed at same place, indicating that EPCs adhered to the damaged endothelial layer. The results showed that CD34 and vWF were colocalized, indicating that EPCs adhered to the inner wall of blood vessels. The staining of vWF in the control group was due to the presence of ECs. Nuclei are stained in blue by DAPI. The white arrows indicate the adhered EPCs at the injury site (*n* = 4). Scale bar = 50 μm.

We established a carotid artery intimal injury model that is shown in Figure 1A to explore the potential interaction between EPCs and VSMCs. The left and right common carotid artery of each rat were used as the experimental group and autologous control, respectively. After 4 weeks, hematoxylin–eosin (HE) staining results indicated that vessel wall was significantly thickened, which revealed the intimal hyperplasia (Figure 1B). To confirm that the intimal injury model was successfully established, we harvested the injury and control vessels right after the surgery. Immunofluorescence staining results showed that the von Willebrand factor (vWF), a marker of the ECs marked in red, was not detected on the inner wall of vessels right after the injury surgery, indicating that the model was successfully established (Figure 1C). In the initial stage of intimal injury, EPCs are mobilized to the injury site; hence, we then examined the adhesion of EPCs 1 h after intimal injury, which were identified by vWF (EC marker) and CD34 (stem cell marker) expression. Due to the balloon injury, the endothelial layer was damaged and did not express vWF, hence, the co-expression of CD34 (red) and vWF (green) indicated the adhesion of EPCs at the injury site (Figure 1D). *In vivo* experiment results suggested that carotid intimal injury caused EPCs homing and adhering to the injury site, which were connected to the exposed VSMCs, which are subjected to physiological stretch at the damaged intima. Hence, we hypothesized that the intercellular communication between EPCs and VSMCs might have an effect on EPC function.

### Stretched VSMCs Promoted the Endothelial Differentiation of Cocultured EPCs

**Figure 2:**
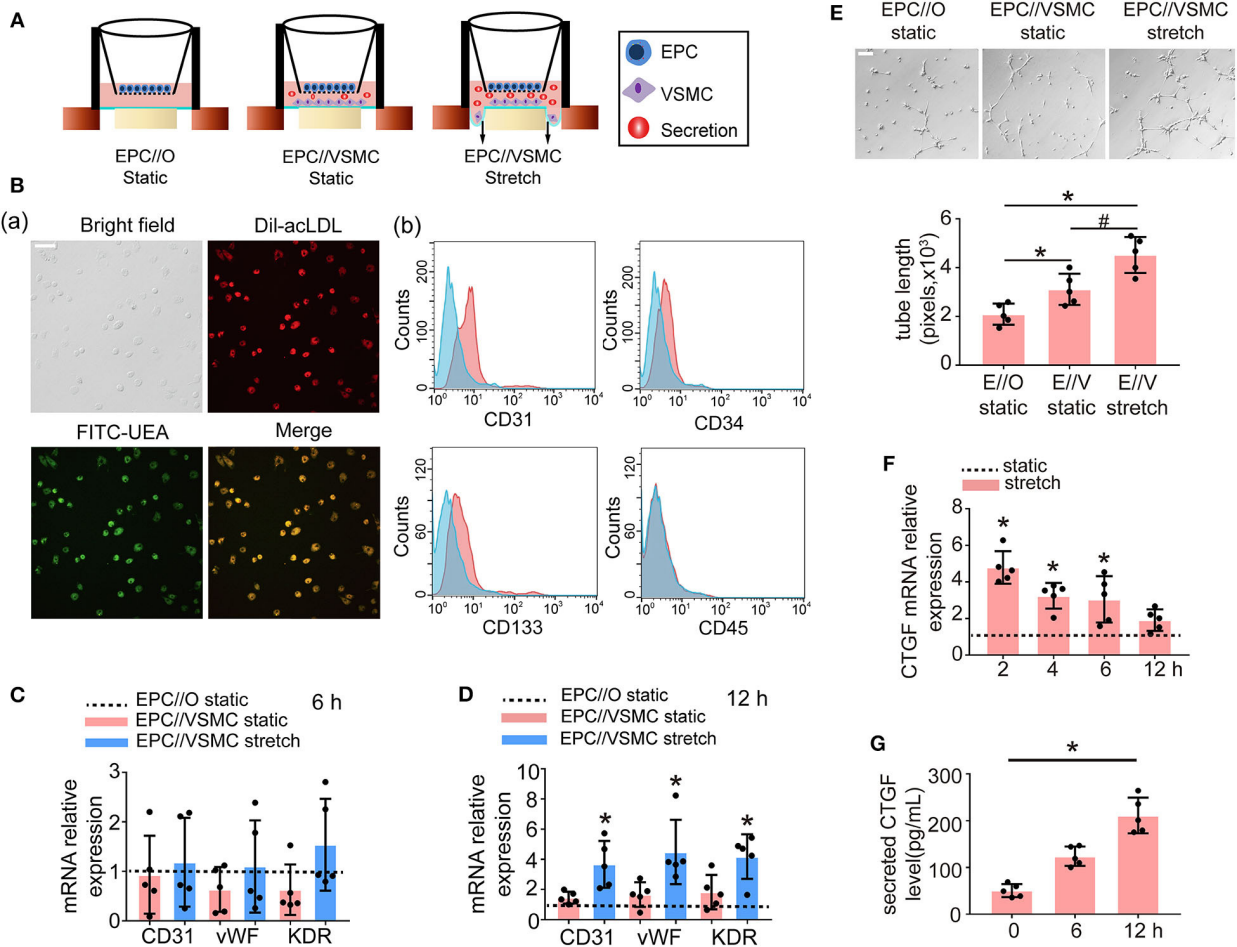
Five percent cyclic stretch induces VSMC-derived CTGF secretion and promotes the differentiation of cocultured EPCs into ECs. **(A)** Schematic diagrams show EPC//VSMC coculture and the cyclic stretch system. **(B)** EPCs showed a spindle-shaped morphology after 8 days. Staining of FITC-UEA-lectin (green) and Dil-acLDL (red) revealed double-positive cells that were identified as EPCs (a). FACS analysis showed that EPCs were positive for the endothelial cell marker CD31 and hematopoietic stem cell markers CD34 and CD133, and they were negative for the leukocyte marker CD45. Controls (blue area) were overlaid on the histogram of each surface antigen (red areas) tested (b). **(C)** The mRNA levels of the EC markers CD31, vWF, and KDR showed no differences in EPCs among different cocultured conditions after 6 h (*n* = 5). Monocultured EPCs under static were used as control groups, shown as the dotted line. **(D)** The mRNA levels of the EC markers CD31, vWF, and KDR were induced in EPCs cocultured with stretched VSMCs after 12 h (*n* = 5). **(E)** EPCs cocultured with stretched VSMCs had an increased tube formation ability after 12 h (*n* = 5). **(F)** QPCR results revealed that CTGF mRNA levels were increased at different time points in stretched VSMCs (*n* = 5). **(G)** The level of CTGF secretion from stretched VSMCs was significantly elevated at 12 h (*n* = 5). For quantitative analysis, five fields per plate were photographed, and tube lengths were measured using Image-Pro Plus software. Scale bar = 100 μm. Values are expressed as the mean ± *SD*. *,^#^*P* < 0.05 compared with the respective control. Statistical analysis was performed with Student's *t*-test and one-way ANOVA for **(C–G)**.

To examine the effect of VSMCs and physiological cyclic stretch on EPC function, an EPC//VSMC coculture with a cyclic stretch application system was used (Figure 2A). EPCs were isolated from SD rat bone marrow by density gradient centrifugation and identified by fluorescence microscopy and flow cytometry. EPCs were characterized as adherent cells that were double positive for Dil-acLDL uptake and FITC-UEA-lectin binding (Figure 2B). Moreover, these cells were found to be positive for CD31, CD34, and CD133 but negative for CD45 by flow cytometric analysis (Figure 2B). VSMCs were identified by immunofluorescent staining with α-smooth muscle actin (SMA), which is shown in green (Supplementary Figure 1).

In the static system, the mRNA levels of the EC markers CD31, vWF, and KDR in EPCs cocultured with VSMCs showed no significant change at 6 h (Figure 2C) or 12 h (Figure 2D), compared to the levels observed in monocultured EPCs. However, once the VSMCs were subjected to 5% cyclic stretch for 12 h, the expression of EC markers in cocultured EPCs was significantly increased as compared with monocultured EPCs (Figure 2D). To determine the role of stretched VSMCs in the angiogenic capability of EPCs, Matrigel tube formation assays were performed. As shown in Figure 2E, the total length of tube-like structures was significantly greater in EPCs cocultured with VSMCs under 5% cyclic stretch than in the static cocultured (E//V, static) or monocultured EPCs (E//O, static) at 12 h. These results indicated that the differentiation of EPCs is regulated by paracrine factors secreted from stretched VSMCs.

In addition, to explore the effect of cyclic stretch on CTGF expression in VSMCs, we detected CTGF expression levels at different time points. The results revealed that both the mRNA and secretion levels of CTGF were elevated in VSMCs exposed to 5% cyclic stretch *in vitro* (Figures 2F,G). The results suggested that CTGF may participate in stretched VSMC-induced EPC differentiation.

### CTGF Induced EPC Differentiation Toward ECs *in vitro*

**Figure 3:**
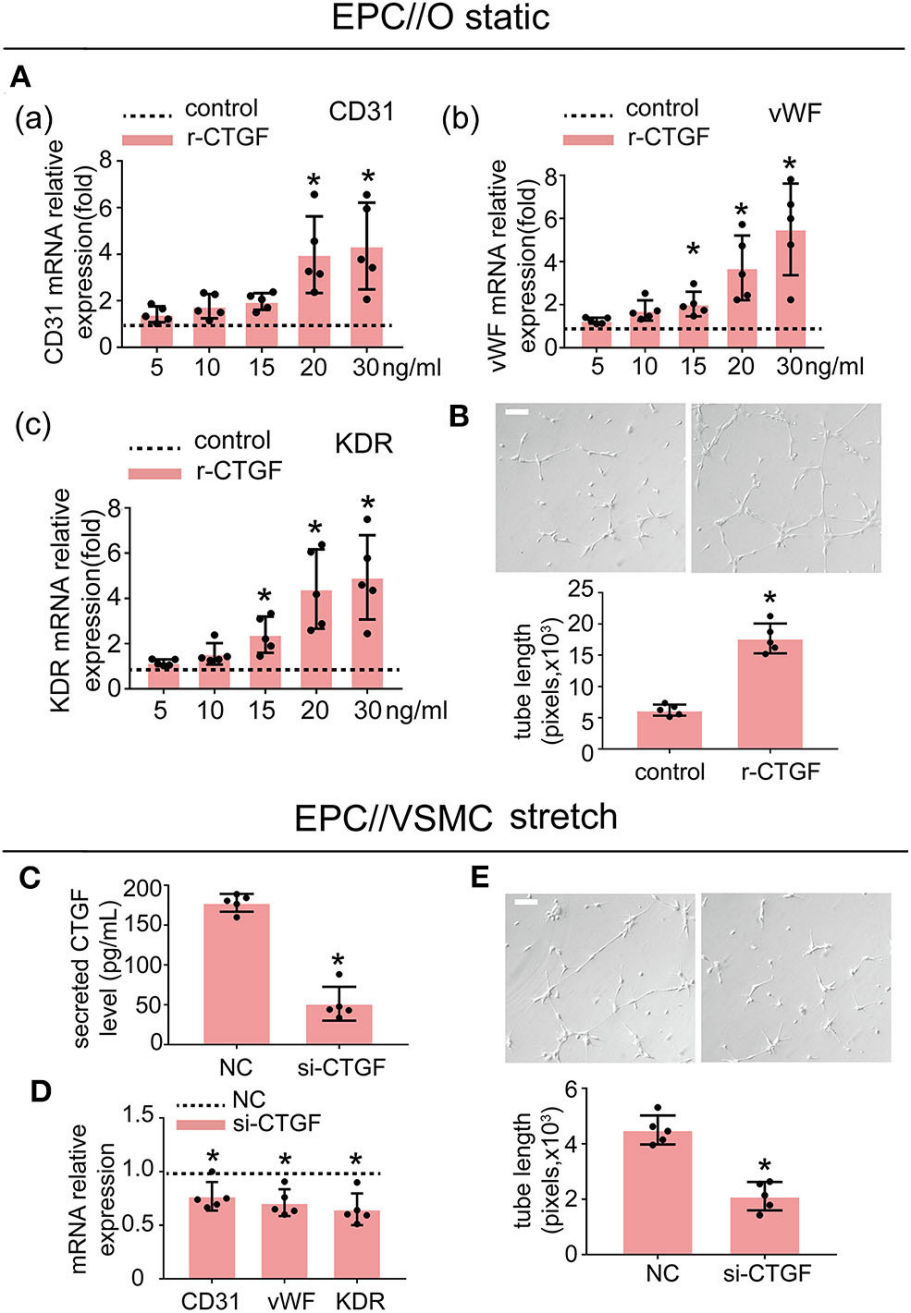
r-CTGF induced EPC differentiation into ECs under static conditions; stretched VSMCs modulate the differentiation of EPCs via CTGF *in vitro*. **(A)** EPCs were treated with different doses of r-CTGF (5, 10, 15, 20, and 30 ng/ml) for 24 h, and the mRNA expression of EC markers CD31, vWF, and KDR were significantly increased at a concentration of 20 ng/ml and 30 ng/ml (*n* = 5). EPCs treated without r-CTGF were used as control groups. **(B)** EPCs treated with r-CTGF (20 ng/ml) for 24 h significantly exhibited increased tube formation (*n* = 5). **(C)** CTGF siRNA was transfected under 5% cyclic stretch conditions, and the CTGF secretion level was significantly decreased in VSMCs. **(D,E)** Transfection with CTGF siRNA under 5% cyclic stretch conditions with cocultured EPCs resulted in the inhibition of EPC differentiation toward ECs and decreased their tube formation (*n* = 5). Scale bar = 100 μm. For quantitative analysis, five fields per plate were photographed, and tube lengths were measured using Image-Pro Plus software. Values are expressed as the mean ± *SD*. *P < 0.05 compared with the respective control. Statistical analysis was performed with Student's *t*-test for **(A–E)**.

To investigate whether CTGF modulates EPC differentiation and angiogenesis, EPCs were treated with r-CTGF. We stimulated EPCs with different doses of r-CTGF protein (0, 5, 10, 15, 20, and 30 ng/ml) for 24 h under static conditions (Figure 3A). Exogenous CTGF at a concentration of 20 ng/ml promoted the expression of the EC markers CD31, vWF, and KDR remarkably in EPCs. Meanwhile, r-CTGF at the concentration of 20 ng/ml promoted EPC tube formation (Figure 3B). To further determine the role of CTGF, its expression was knocked down in VSMCs by CTGF-specific siRNA. After transfected with CTGF siRNA for 48 h, VSMCs were then subjected to cyclic stretch. The effect of CTGF siRNA on the secretion level of CTGF was significantly decreased in VSMCs (Figure 3C). CTGF knockdown in stretched VSMCs could reverse EPC differentiation and angiogenesis capabilities (Figures 3D,E) in comparison with the control group. Overall, the results indicated that CTGF plays a key role in stretched VSMC-induced EPC differentiation *in vitro*.

### The Potential Downstream Targets of CTGF Were Predicted by Ingenuity Pathway Analysis

**Figure 4:**
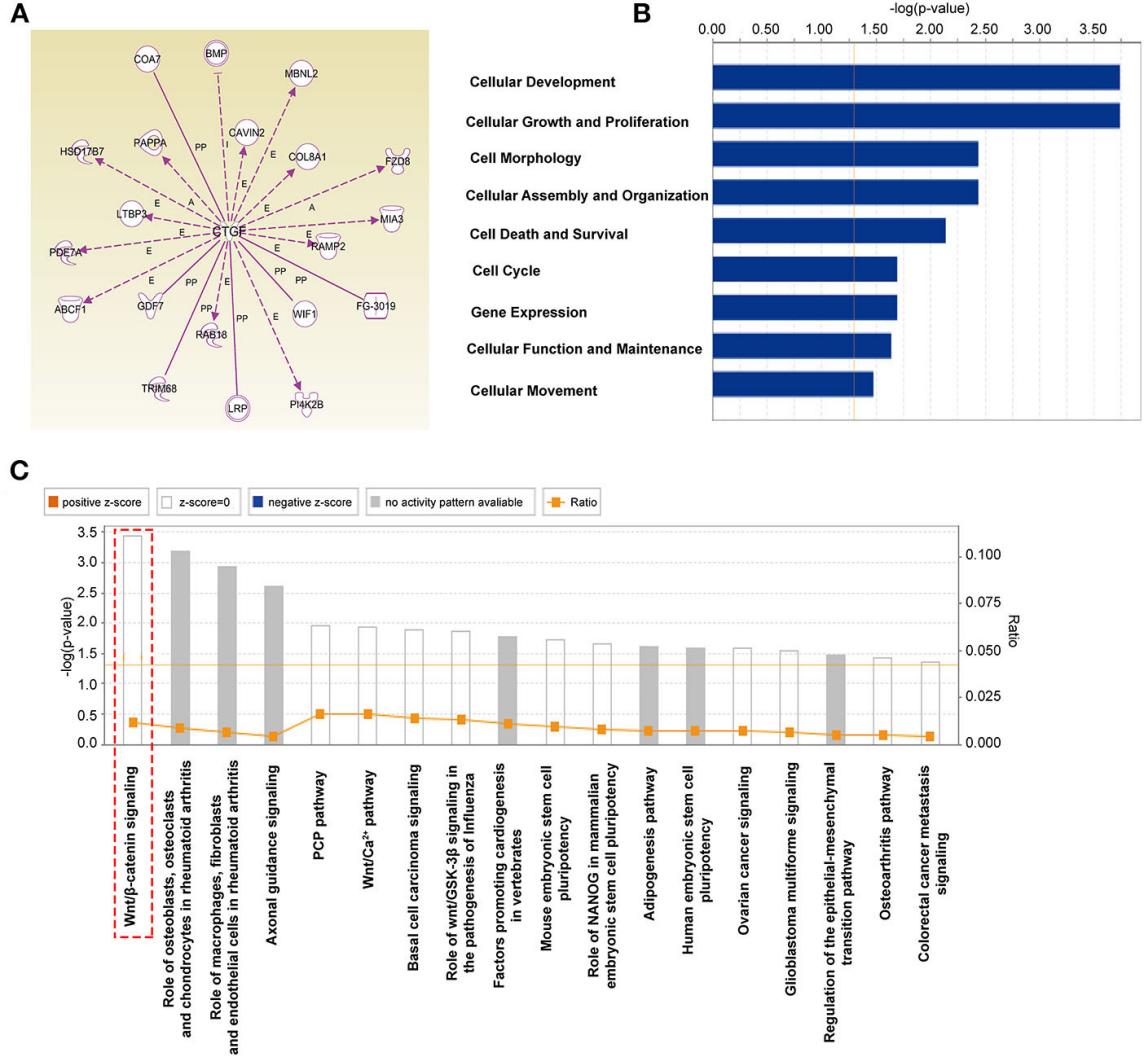
The potential downstream targets of CTGF that were predicted by IPA are shown. **(A)** Molecules predicted to interact with CTGF were determined using IPA software. IPA analysis showed that downstream targets are involved in molecular and cellular functions **(B)** and canonical pathways **(C)**.

CTGF is essential for the differentiation of EPCs; however, the underlying mechanism involved remains unclear and needs to be further studied. By using ingenuity pathway analysis (IPA) with high prediction criteria, the top 20 molecules that are potentially downstream of CTGF were selected (Figure 4A). Then, IPA analysis allowed further insights into the cellular functions (Figure 4B) and canonical pathways (Figure 4C) of the top 20 genes. The top molecular and cellular function was cellular development, and the top canonical pathway that was identified was the Wnt/β-catenin signaling pathway, which plays an essential role in developmental decisions and regulating progenitor cell fate during embryonic development. According to reports, FZD8, a member of Frizzled receptors located on the cell membrane, functions as a key component of the Wnt signaling pathway (Chakravarthi et al., 2018; Murillo-Garzon et al., 2018). When Wnt proteins bind to transmembrane FZD8 receptors and coreceptors, β-catenin-dependent signals can be activated (Murillo-Garzon et al., 2018). Thus, we hypothesized that FZD8 and β-catenin, which are key regulators of the Wnt/β-catenin signaling pathway, might participate in the process of CTGF-induced EPC differentiation.

### CTGF Regulated FZD8/β-catenin Expression in EPCs

**Figure 5:**
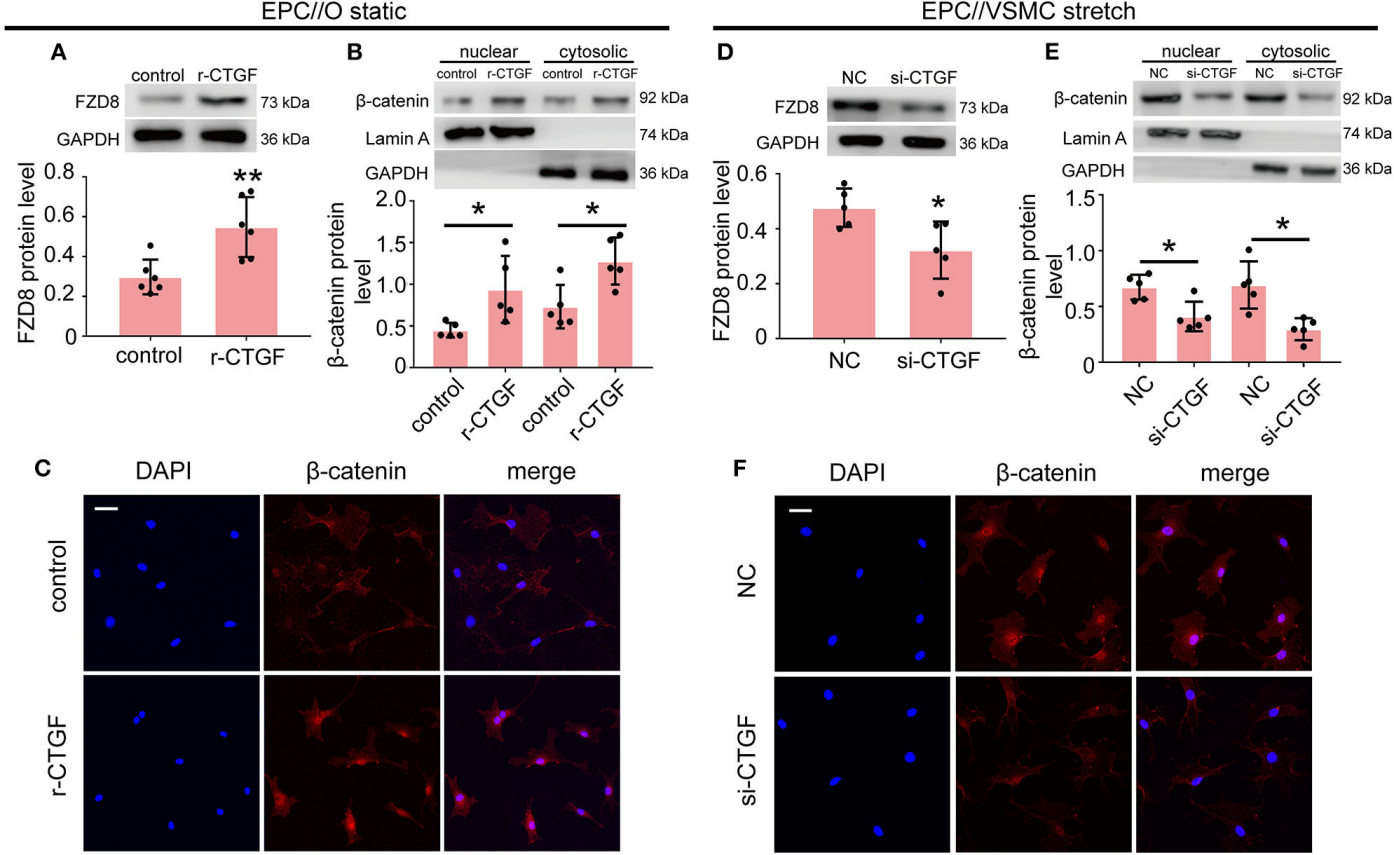
CTGF regulates FZD8/β-catenin expression in EPCs. **(A)** The FZD8 protein level was markedly induced in EPCs treated with r-CTGF (20 ng/ml) for 24 h in comparison with the control (*n* = 5). **(B,C)** Both Western blotting and immunofluorescent staining showed upregulated β-catenin protein levels in the nuclei and cytosol of EPCs stimulated with r-CTGF. **(D)** Transfection with CTGF siRNA in VSMCs under 5% cyclic stretch conditions repressed the FZD8 protein level in cocultured EPCs (*n* = 5), and **(E,F)** nuclear and cytosolic β-catenin protein expression was also suppressed in cocultured EPCs. Scale bar = 50 μm. Values are expressed as the mean ± *SD*. *P < 0.05 compared with the control. Statistical analysis was performed with Student's *t*-test for **(A,B,D,E)**.

To further determine the effect of CTGF on FZD8 and β-catenin expression, EPCs were treated with r-CTGF *in vitro*. Western blot results revealed that r-CTGF induced the expression of FZD8 (Figure 5A). Moreover, the protein level of β-catenin was increased in both nuclear and cytosolic lysates from EPCs (Figure 5B). As shown in Figure 5C, r-CTGF treatment leads to significant increases of β-catenin in both the cytoplasm and the nucleus of EPCs. To further determine the role of CTGF under cyclic stretch conditions, its expression was knocked down in VSMCs by CTGF-specific siRNA. VSMCs were treated with CTGF siRNA for 48 h and then subjected to cyclic stretch. In contrast to the effect of r-CTGF, knockdown of CTGF in VSMCs not only suppressed FZD8 (Figure 5D) and β-catenin (Figure 5E) protein expression but also decreased the expression of β-catenin in both the cytoplasm and the nucleus of EPCs (Figure 5F). Taken together, these findings suggest that CTGF may modulate EPC differentiation and angiogenesis via the FZD8 and β-catenin pathways.

**Figure 6:**
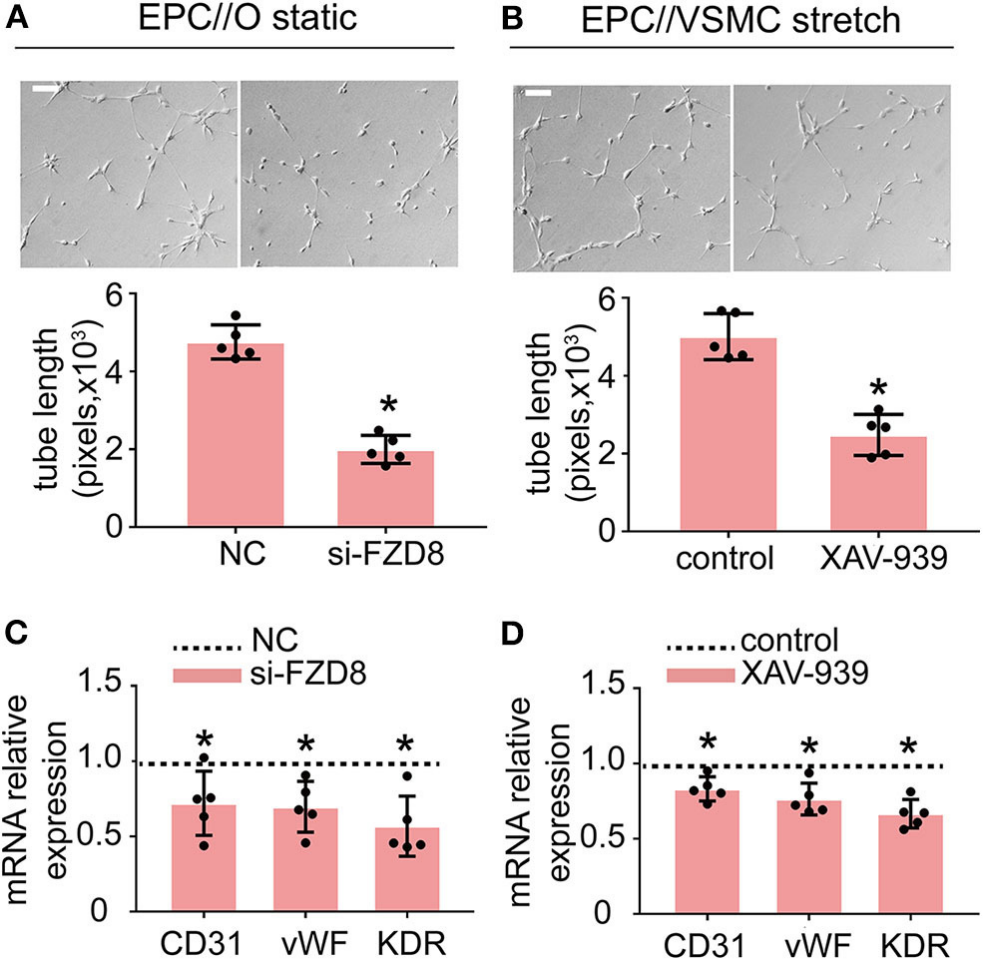
Treatment with FZD8 siRNA and XAV-939 decreased EPC differentiation and tube formation. **(A,B)** EPCs transfected with FZD8 siRNA for 24 h exhibited significantly decreased tube formation and decreased expression of EC markers (CD31, vWF, and KDR) (*n* = 5). **(C,D)** Treatment with XAV-939 (5 μM) decreased tube formation and inhibited EPC differentiation toward ECs (*n* = 5). Scale bar = 100 μm. For quantitative analysis, five fields per plate were photographed, and tube lengths were measured using Image-Pro Plus software. Values are expressed as the mean ± *SD*. *P < 0.05 compared with the control. Statistical analysis was performed with Student's *t*-test for **(A–D)**.

To better understand the role of FZD8 and Wnt signaling in regulating EPC differentiation, EPCs were treated with an FZD8-specific siRNA and with XAV-939 (specific inhibitor of Wnt signaling), respectively. The effect of the siRNA treatments on FZD8 mRNA levels was assessed (Supplementary Figure 2). The results indicated that FZD8 knockdown in EPCs could decrease angiogenic ability (Figure 6A) and could decrease EPC differentiation into the EC lineage (Figure 6B). To confirm the role of Wnt signaling in cyclic stretch-induced actions of CTGF on EPCs, the inhibitor of Wnt signaling, XAV-939, was used to treat cocultured EPCs at the concentration of 5 μM. XAV-939 treatment remarkably inhibited EPC tube formation (Figure 6C). Meanwhile, XAV-939 treatment decreased the expression of the EC markers CD31, vWF, and KDR in EPCs (Figure 6D).

### CTGF Promoted the Differentiation of EPCs *in vivo*

**Figure 7:**
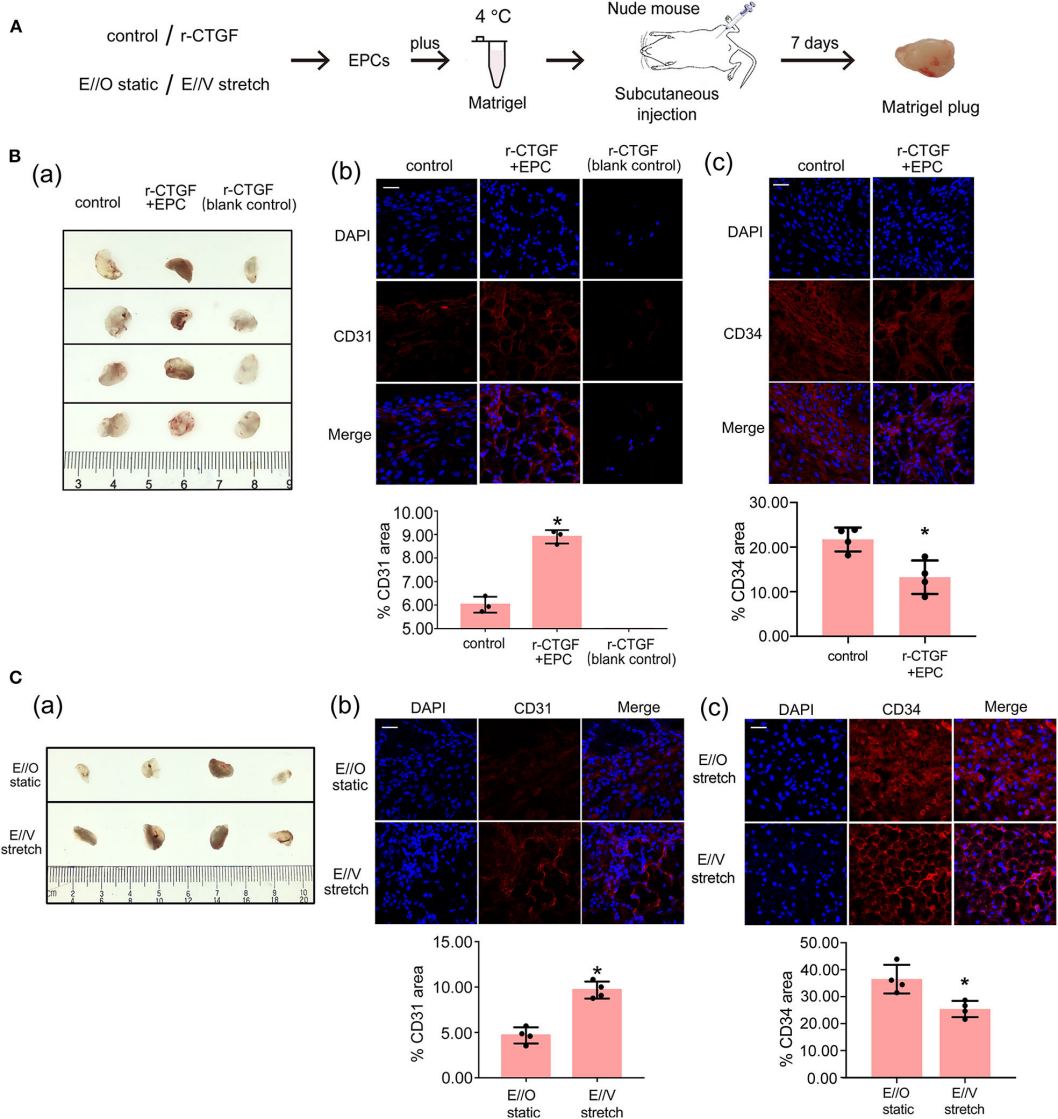
The Matrigel plug assay results shows that r-CTGF or stretched VSMCs promote the differentiation of EPCs *in vivo*. **(A)** Transplantation scheme is shown for the *in vivo* Matrigel plug assay. **(B)** r-CTGF promoted angiogenesis of EPCs in *vivo*. Immunofluorescent staining for CD31 revealed increased arterial endothelium formation in the Matrigel with r-CTGF-treated EPCs. In addition, few cells invaded the plug in the Matrigel that was injected r-CTGF in the absence of EPCs. The stem cell marker CD34 staining results showed that protein expression levels in EPCs treated with r-CTGF were significantly reduced compared to the control group. **(C)** Stretched VSMCs promoted the ability of EPCs to form tubes *in vivo*. Immunofluorescent staining for CD31 revealed that CD31 expression in EPCs cocultured with stretched VSMCs was significantly increased. EPCs that were cocultured with VSMCs that were exposed to cyclic stretch in Matrigel plug expressed lower level of CD34 compared with monocultured under static conditions (*n* = 4). Scale bar = 50 μm. Values are expressed as the mean ± *SD*. *P < 0.05 compared with the control. Statistical analysis was performed with Mann–Whitney *t*-test for **(B,C)**.

To examine the role of CTGF in EPC differentiation *in vivo*, the Matrigel plug assay was performed. In this assay, EPCs with or without r-CTGF treatment were mixed with Matrigel and subcutaneously injected into the flank of nude mice, and Matrigel supplemented only with r-CTGF in the absence of EPCs was used as a blank control. On day 7, mice were sacrificed, and Matrigel plugs were harvested (Figure 7A). Abundant vessel formation was shown in the plugs of EPCs treated with r-CTGF compared with what was observed in the controls. In addition, few cells invaded the plug in the Matrigel that was injected r-CTGF in the absence of EPCs. The Matrigel plugs were embedded in paraffin to enable histological analysis and the level of EPC differentiation was detected using an antibody against the EC marker CD31. We observed a significant increase in CD31 staining in the group of r-CTGF-treated EPCs, and there was no CD31 staining in the sections of plugs mixed with r-CTGF and no EPCs (Figure 7B). To further demonstrate that r-CTGF promoted endothelial differentiation of EPCs *in vivo*, we also performed the stem cell marker CD34 immunofluorescence staining. CD34 staining results showed that protein expression levels in EPCs treated with r-CTGF were significantly reduced compared to the control group (Figure 7B). The results indicated that CTGF enhanced the differentiation of EPCs into ECs *in vivo*.

Furthermore, to investigate how VSMCs and stretch affect EPC differentiation, another Matrigel plug assay was also performed. EPCs were monocultured under static conditions or cocultured with VSMCs that were subjected to cyclic stretch at 5% stretch magnitude and 1.25 Hz frequency. After 12 h, EPCs under different conditions were harvested and mixed with growth factor-reduced Matrigel. Matrigel was injected subcutaneously into the flank of nude mice. On day 7, the Matrigel plugs were harvested after mice were sacrificed. The results show that there is a significant increase in CD31 staining and decrease in CD34 in the plugs mixed with cocultured EPCs under cyclic stretch conditions in comparison to the group with monocultured EPCs under static conditions; these results indicate that VSMCs and stretching prompt the differentiation of EPCs into the endothelial lineage (Figure 7C).

### CTGF Promoted Reendothelialization Capacity of EPCs After Intimal Injury *in vivo*

**Figure 8:**
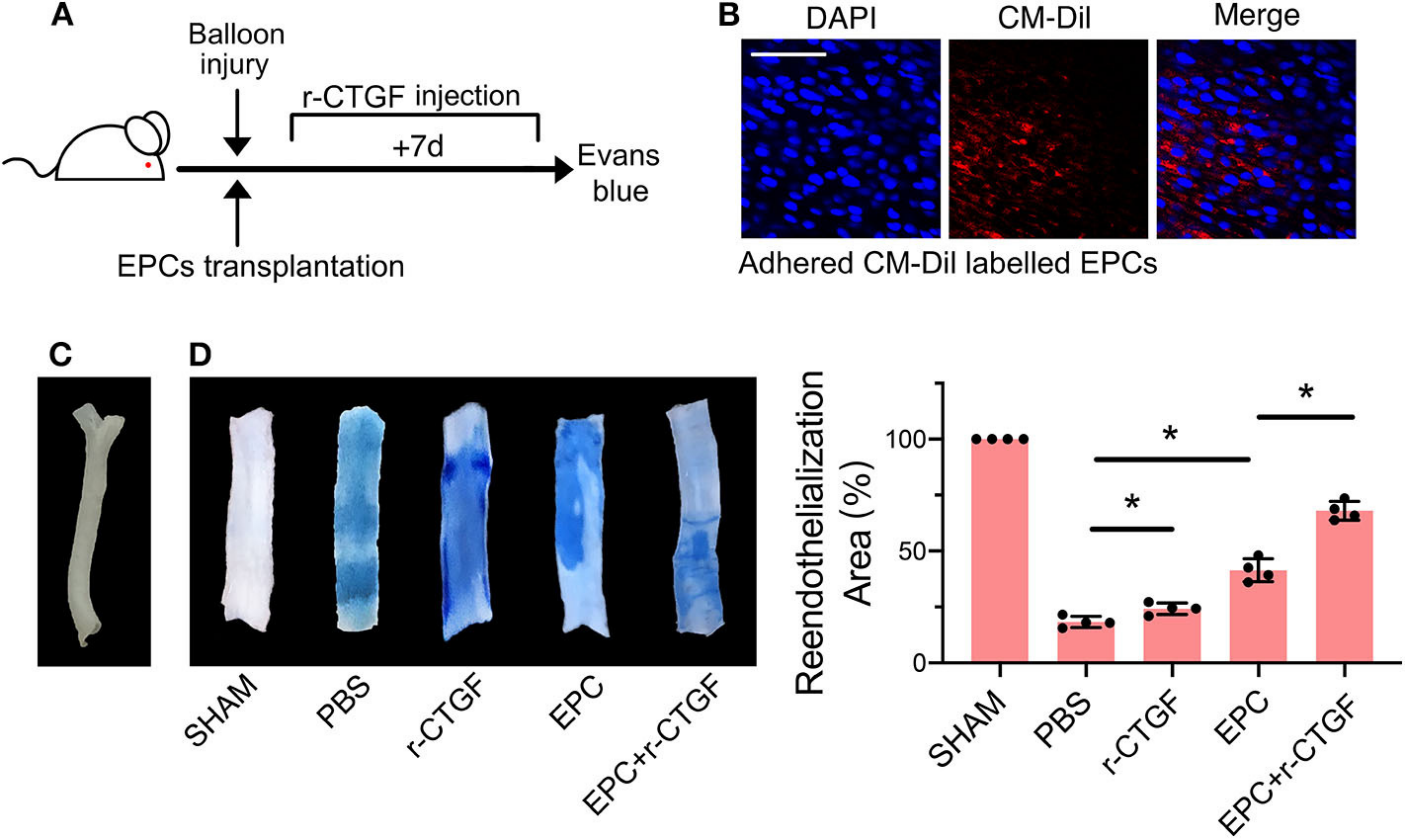
EPC and r-CTGF promoted vascular intimal injury repair. **(A)** Schematic diagrams of the animal experimental protocol. **(B)** CM-Dil-labeled EPCs (red) adhered to the vascular lesion in situ. **(C)** The image of rat left carotid artery. **(D)** Intimal injury site was incubated with or without EPCs for 25–30 min, and injection of r-CTGF (2 μg/kg/day) promoted vascular repair. Reendothelialization area was observed by Evans blue staining after 7 days of the surgery. The percentage of the white area indicated the degree of reendothelialization. Scale bar = 50 μm. Values are expressed as the mean ± *SD*. *P < 0.05 compared with the control. Statistical analysis was performed with Mann-Whitney *t*-test for D.

To further verify the effect of CTGF on EPC differentiation after intimal injury, the rat carotid intimal injury model was established. After carotid intimal injury, CM-Dil-labeled EPCs were incubated at the fresh injury site. The adherent EPCs directly contacted with VSMCs, and meanwhile, both cells were under cyclic stretch. The degree of reendothelialization after vascular injury is widely recognized and used in the detection of differentiation of EPCs and the repair of intimal injury by Evans blue dye. The flow diagram of the experimental protocol was shown in Figure 8A. Immunofluorescence staining results indicated that CM-Dil-labeled EPCs adhered to the vascular lesion *in situ* (Figure 8B). The rat left carotid artery without surgery was imaged in Figure 8C. Evans blue staining was used to detect reendothelialization after intimal injury. Compared with the sham operation group, the experimental group treated with PBS injection only had the largest blue area. The blue area decreased slightly after systemic injection of r-CTGF, indicating an increase of vascular reendothelialization. Furthermore, after intimal injury, local incubation with EPCs significantly increased intimal repair, and tail vein injection of r-CTGF for 7 days significantly promoted reendothelialization capacity of EPCs and upregulated reendothelialization area (Figure 8D).

## Discussion

In this study, we characterized the so far unknown synergistic effect of physiological cyclic stretch and communication with neighboring VSMCs on endothelial differentiation of EPCs and their angiogenic activity. Our results highlight and help to better understand the role of mechanical stretch and regulation of intracellular communication in vascular repair, which indicate that cyclic stretch induces VSMC-derived CTGF secretion, which in turn activates FZD8 and β-catenin to promote both the endothelial differentiation of cocultured EPCs and angiogenesis.

Increasing reports have shown that EPCs, which quiescently reside in physiological bone marrow and can be recruited to sites of injury, are important for maintaining endothelial integrity and take part in the process of re-endothelization, neovascularization, and wound healing by differentiating into mature ECs and secreting angiogenic factors when endothelial injury occurs (Hristov et al., 2003; Hur et al., 2004; Zhang et al., 2014). Previous studies have revealed that the differentiation fate of EPCs is affected by many factors, such as mechanical forces (including shear stress and cyclic stretch) and angiogenic factors (such as VEGF and CCN61) (Yu et al., 2010; Cheng et al., 2014; Li et al., 2017, 2018). Mesenchymal stem cells promote the endothelial differentiation of EPCs with the upregulation of CD31 and vWF (Ge et al., 2018). It has been reported that smooth muscle progenitor cells were reported to promote the neovascularization of EPCs and enhance the efficiency of EPC therapy during the wound-healing process (Joo et al., 2014; Foubert et al., 2015). Shudo et al. showed that the normal interactions between VSMCs and EPCs increased functional microvasculature and enhanced myocardial function in a rodent ischemic cardiomyopathy model (Shudo et al., 2013). However, the influences of neighboring VSMCs and physiological stretch on EPC functions are unknown. In this study, we developed a coculture system that assured intimate cellular communication between EPCs and VSMCs and included the application of physiological mechanical stretching. The present findings show that VSMCs facilitate EPC differentiation into ECs that have elevated expression of EC markers (CD31, vWF, and KDR) and increased angiogenic ability in the presence of mechanical stretch, indicating the importance of the synergistic effect of VSMCs and mechanical forces on EPC function.

In the present study, the Flexcell FX-5000T Strain Unit was applied to generate mechanical stretch *in vitro*. The 5% cyclic stretch (mimicking the physiological cyclic stretch) magnitude is based on previous clinical ultrasound data, which indicated that the large artery dilates by as much as 5% during cyclic stretch in normotension (Asanuma et al., 2003; Maul et al., 2011). Furthermore, the frequency used in the present study simulates the adult heart rate (70–80 beats) (Wachowiak et al., 2016), and some studies on rat VSMC cultures use an even lower frequency. The magnitude and frequency used here have also been used in our previous publications (Qi et al., 2016; Yao et al., 2017).

It has been shown that mechanical stretching increased the amount of CTGF secretion in anterior cruciate ligament-derived cells (Miyake et al., 2011), indicating that CTGF is a mechano-sensitive molecule. Blomme et al. reported that valvular interstitial cells showed the significant upregulation of CTGF at 1.15 Hz (heart frequency) and 14% elongation mechanical stretch, which was promoted through RhoC and MEK/Erk signaling pathways (Blomme et al., 2019). Additionally, in our previous study, we found that pathological cyclic stretch induces vascular remodeling by promoting VSMC proliferation via the miR-19b-3p/CTGF pathway (Wang et al., 2019).

Accumulating evidence indicates that CTGF, as a potent angiogenic inducer, supports cell metastasis, invasion, and angiogenesis in several tumor cells, and it induces tube formation in ECs (Kubota and Takigawa, 2007). It suggests that there is probably an interaction between angiogenesis of EPCs and CTGF. Our previous results indicated that 5% cyclic stretch induced the expression of CTGF in VSMCs; hence, in the current study, we aimed to investigate whether stretch-induced CTGF plays a role in EPC function. The results showed that r-CTGF significantly enhanced the expression of endothelial markers at the mRNA level and enhanced tube formation, whereas VSMCs transfected with a CTGF-specific siRNA suppressed this expression. Meanwhile, by using IPA bioinformatics analysis, FZD8 was found to be a putative downstream target regulated by CTGF.

FZD8 and β-catenin are key regulators in the Wnt/β-catenin signaling pathway that have essential roles in the regulation of progenitor cell fate, adult tissue homeostasis, proliferation during embryonic development, and developmental decisions (Majidinia et al., 2018). For example, ATP activates Wnt/β-catenin signaling in mesenchymal stem cells (MSCs), which contributes to neuronal differentiation by inducing the expressing of neuronal markers, Tuj1 and NeuN (Tu et al., 2014). Attenuation of Wnt/β-catenin signaling by exposure to the epidermal growth factor receptor is required for hair follicle development (Tripurani et al., 2018). In addition, during retinal neovascularization in oxygen-induced retinopathy, the Wnt/β-catenin signaling pathway had an important role in EPC recruitment (Liu et al., 2013). In our study, the positive effect of CTGF on FZD and β-catenin expression was detected. Therefore, CTGF may modulate the differentiation fate and angiogenic ability of EPCs by activating the FZD8/β-catenin pathway. Moreover, to better understand the regulation mechanism, we further detected the FZD8 mRNA level of EPCs after the r-CTGF stimulation. Results showed that the mRNA level was significantly increased by r-CTGF, which suggested that FZD8 was regulated by CTGF via transcriptional regulation (Supplementary Figure 3). We also further examined the effect of FZD8 loss of function or Wnt signaling in EPCs to confirm whether they are important for EPC differentiation. Our results indicated that FZD8/β-catenin is vital for EPC differentiation and angiogenic activities.

**Figure 9:**
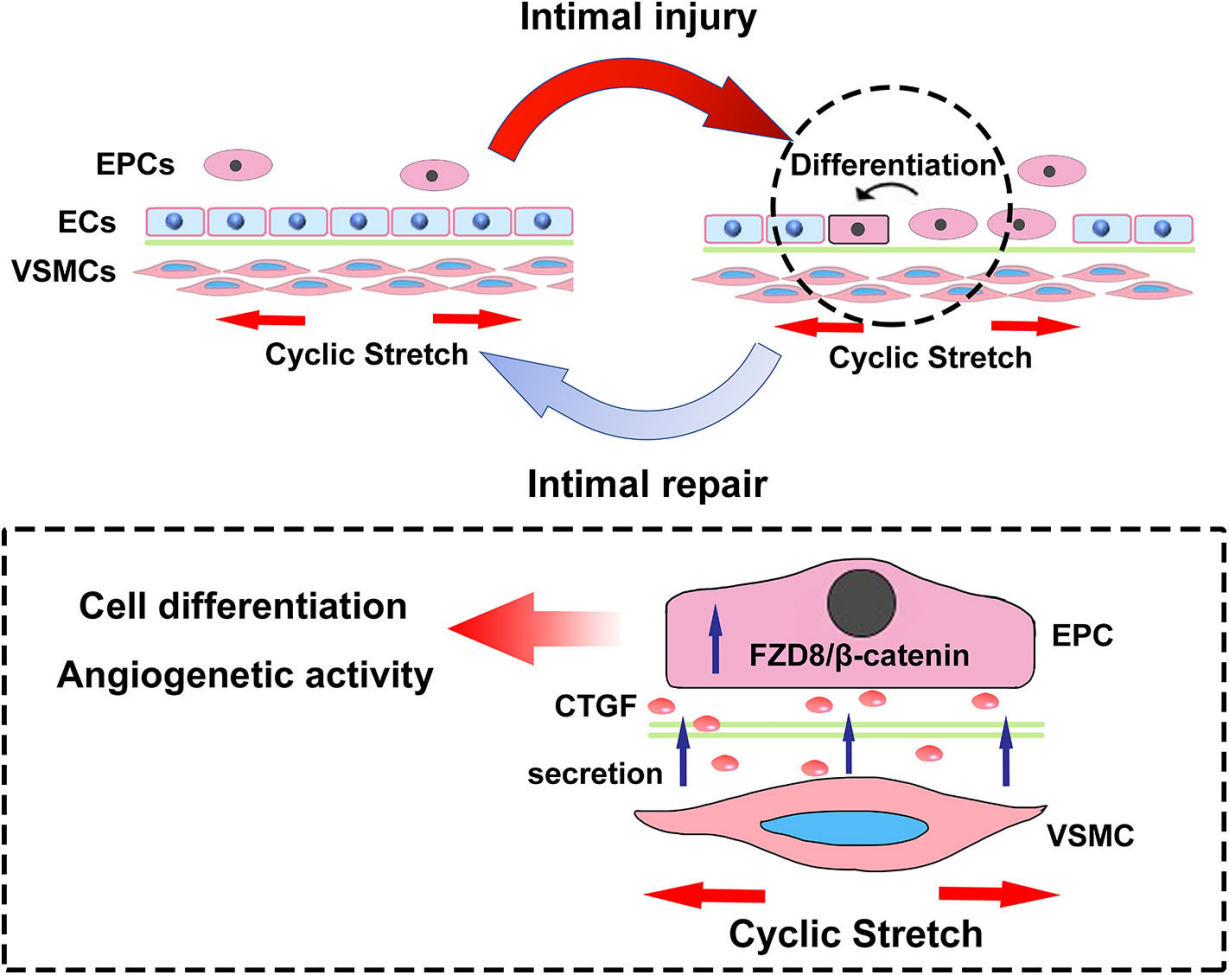
The scheme here depicts the microenvironmental roles of VSMCs under cyclic stretch in EPC differentiation and angiogenesis during vascular repair. Cyclic stretch promotes the secretion of CTGF from VSMCs, which subsequently activates the FZD8/β-catenin signaling pathway in EPCs, and increased β-catenin nuclear translocation eventually induces the differentiation and angiogenic abilities of EPCs.

In conclusion, the present results revealed critical microenvironmental roles for VSMCs in the differentiation and angiogenesis process of EPCs during vascular injury. As is shown in Figure 9, physiological cyclic stretch (5%) increased the secretion of CTGF from VSMCs. Secreted CTGF activated FZD8 in EPCs, which subsequently promoted β-catenin nuclear translocation from the cytoplasm and finally encouraged EPC differentiation fate toward ECs with enhanced angiogenic ability and reendothelialization capacity. *In vivo* study also indicated that stretched VSMCs induced cocultured EPC differentiation toward ECs; furthermore, r-CTGF enhanced angiogenesis and improved EPC reendothelialization capacity. The CTGF/FZD8/β-catenin signaling axis may become a promising therapeutic target for vascular repair.

## Data Availability Statement

The original contributions presented in the study are included in the article/Supplementary Materials, further inquiries can be directed to the corresponding author.

## Ethics Statement

The animal study was reviewed and approved by the Animal Research Committee of Shanghai Jiao Tong University.

## Author Contributions

JY: planned, performed, and analyzed animal studies and some of the cell and microscopy studies, prepared figures, and wrote part of the manuscript. W-BW: planned, performed, and analyzed the cell and microscopy studies, prepared figures, and wrote part of the manuscript. Y-JF: contributed to the immunofluorescence staining studies and the supplementary experiments in this project. HB: contributed to some of the animal studies. NL: contributed to the initial studies in this project. Q-PY, Y-LH, Z-LJ, Y-XQ, and YH: contributed to experimental facilities and reagents in this project. YH: planned and supervised the study and contributed to the writing of the final manuscript. The first draft of the manuscript was written by W-BW, JY, and YH. All authors contributed to the study conception and design, read, and approved the final manuscript.

## Conflict of Interest

The authors declare that the research was conducted in the absence of any commercial or financial relationships that could be construed as a potential conflict of interest.

## Supplementary Material

Supplementary Figure 1 VSMC identities are confirmed. Staining with α-SMA (green) revealed that the positively stained cells were VSMCs. Scale bar = 100 μm.

Supplementary Figure 2 Transfection with an FZD8 siRNA significantly decreased the FZD8 mRNA level (*n* = 3). Values are expressed as the mean ± *SD*. ^*^*P* < 0.05 compared with the control.

Supplementary Figure 3 The mRNA expression level of FZD8 in EPCs was stimulated by r-CTGF (*n* = 4). Values are expressed as the mean ± *SD*. ^*^*P* < 0.05 compared with the control.

## Abbreviations

CTGF: connective tissue growth factor
DMEM: Dulbecco's modified Eagle medium
EPCs: endothelial progenitor cells
ECs: endothelial cells
EGM-2: endothelial growth medium-2
ELISA: enzyme-linked immunosorbent assay
FBS: fetal bovine serum
IPA: ingenuity pathway analysis
PET: polyethylene terephthalate
SD rats: Sprague–Dawley rats
siRNA: small interfering RNA
VSMCs: vascular smooth muscle cells.
