# Supplementary figures and images for: Cyclic Stretch Induces Vascular Smooth Muscle Cells to Secrete Connective Tissue Growth Factor and Promote Endothelial Progenitor Cell Differentiation and Angiogenesis
Source: Front Cell Dev Biol. 2020 Dec 9;8:606989. doi: 10.3389/fcell.2020.606989 (PMC7755638; doi:10.3389/fcell.2020.606989)

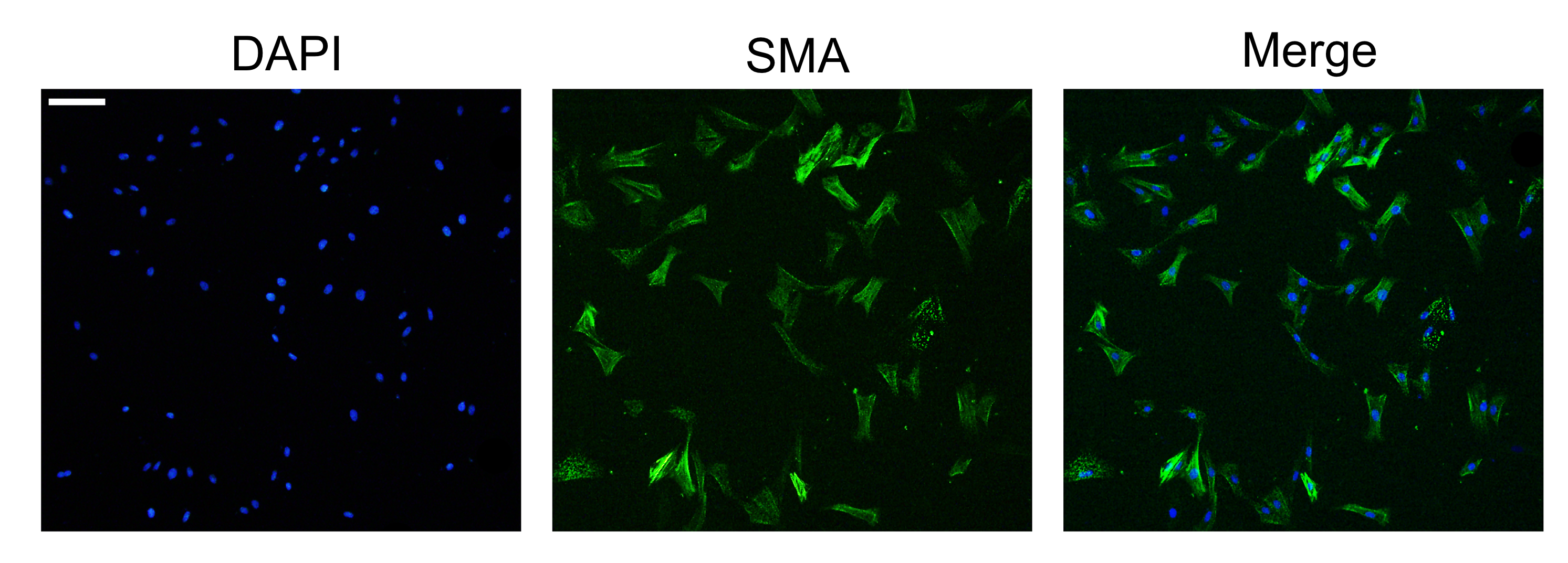

Supplement: Supplementary Figure 1 — VSMC identities are confirmed. Staining with α-SMA (green) revealed that the positively stained cells were VSMCs. Scale bar = 100 μm. [file Image_1.TIF]

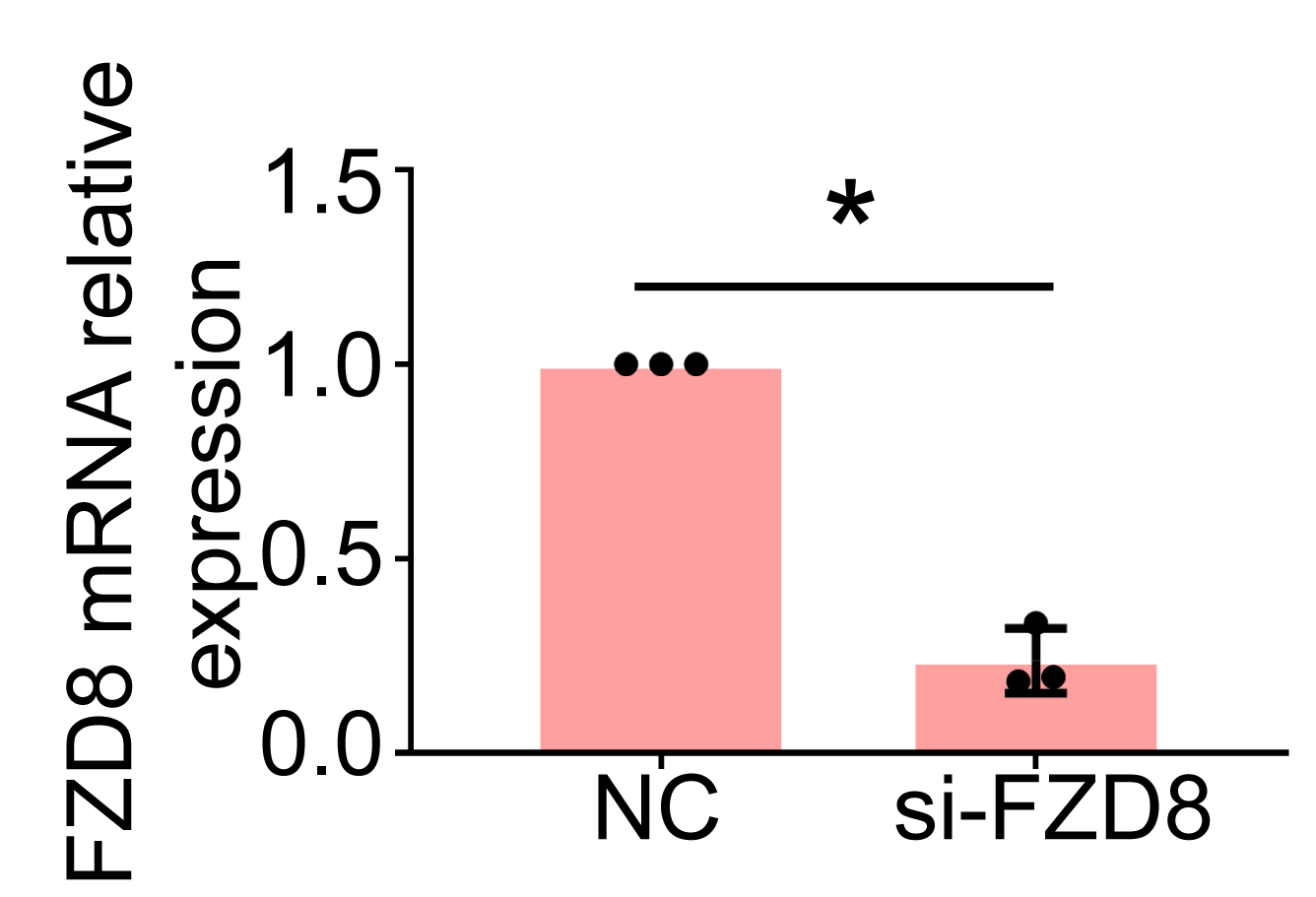

Supplement: Supplementary Figure 2 — Transfection with an FZD8 siRNA significantly decreased the FZD8 mRNA level (n = 3). Values are expressed as the mean ± SD. *P < 0.05 compared with the control. [file Image_2.TIF]

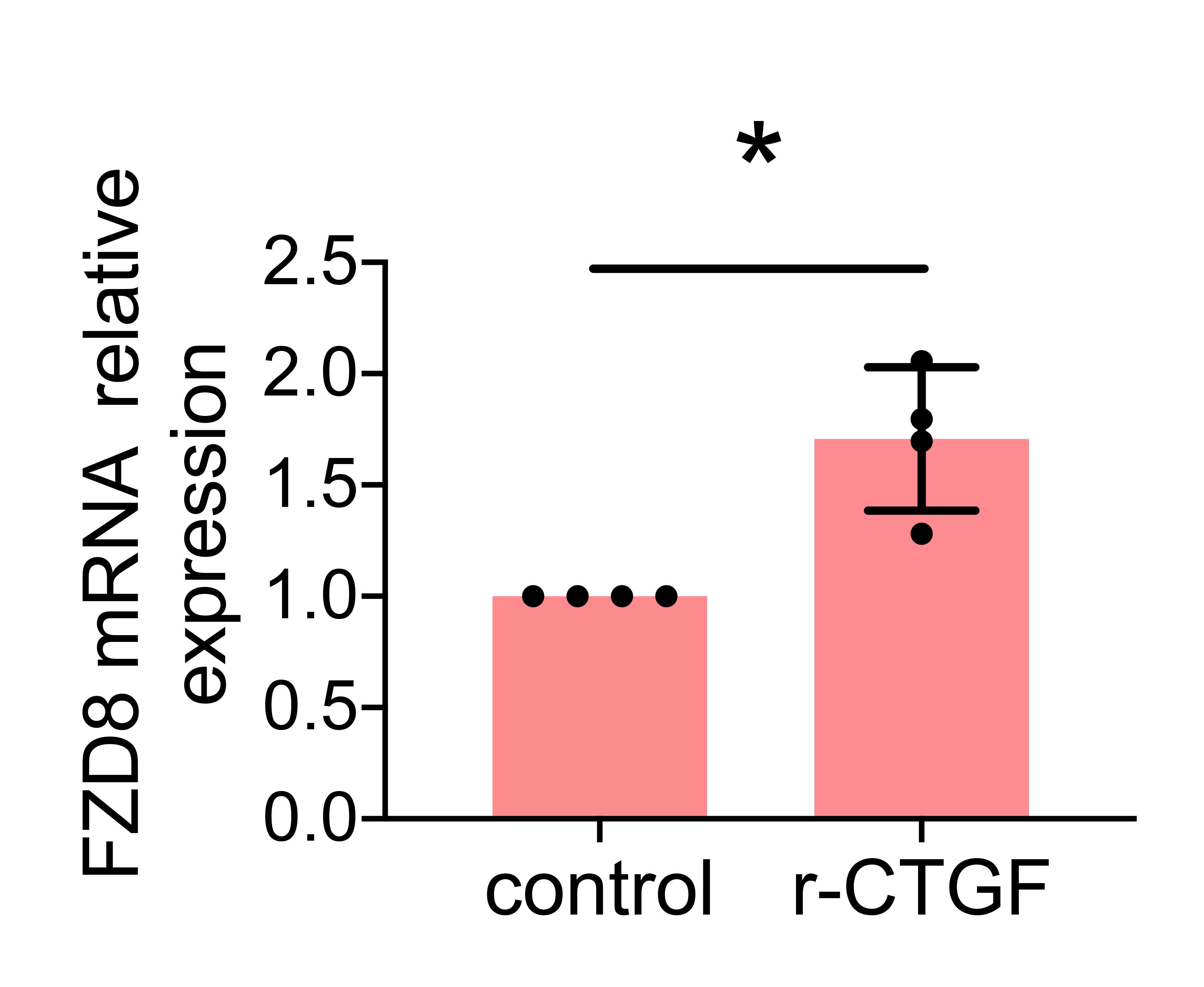

Supplement: Supplementary Figure 3 — The mRNA expression level of FZD8 in EPCs was stimulated by r-CTGF (n = 4). Values are expressed as the mean ± SD. *P < 0.05 compared with the control. [file Image_3.TIF]
